# Behavioral and Cognitive Interventions With Digital Devices in Subjects With Intellectual Disability: A Systematic Review

**DOI:** 10.3389/fpsyt.2021.647399

**Published:** 2021-04-13

**Authors:** Marta Torra Moreno, Josefa Canals Sans, Maria Teresa Colomina Fosch

**Affiliations:** ^1^Jeroni de Moragas Private Fundation, Tarragona, Spain; ^2^Jeroni de Moragas Association, Tarragona, Spain; ^3^Department of Psychology, Research Center for Behavioral Assessment (CRAMC), Rovira i Virgili University, Tarragona, Spain; ^4^Research Group in Neurobehavior and Health (NEUROLAB), Tarragona, Spain

**Keywords:** intellectual disability, cognitive intervention, behavioral intervention, digital devices, computer, handheld, systematic review

## Abstract

In recent years, digital devices have been progressively introduced in rehabilitation programs and have affected skills training methods used with children and adolescents with intellectual disabilities (ID). The objective of this review is to assess the effects of the use of digital devices on the cognitive functions and behavioral skills in this population, and to acknowledge their potential as a therapeutic tool. Electronic databases were analyzed until February 2020 using search formulas with free terms related to ID and the use of digital systems with children or adolescents. The risk of bias in randomized controlled trials was assessed by means of the modified Cochrane Collaboration tool and the quality level of the non-randomized studies was assessed using the Newcastle-Ottawa Scale. Forty-four studies were analyzed, most of which were categorized as low quality. Of the executive function studies analyzed, 60% reported significant improvements, most commonly related to working memory. Within the cognitive skills, 47% of the studies analyzed reported significant improvements, 30% of them in language. Significant improvements in the social (50%) and behavioral domains (30%) were also reported. These results suggest that digital interventions are effective in improving working memory and academic skills, and positively affect both the social and behavioral domains. Little information has been published regarding the duration of the effects, which could be limited in time. Further research is necessary to assess long-term effectiveness, the influence of comorbidities, and the effects on subjects with severe ID. The inclusion of smartphones and special education centers is also necessary.

## Introduction

The Diagnostic and Statistical Manual of the American Psychiatric Association, Fifth Edition (DSM-5) (1), defines the concept of “intellectual disability” (ID) as a “disorder that begins during the developmental period and it includes limitations in intellectual functioning and also adaptive behavior in the conceptual, social and practical domains.” The meta-analysis of McKenzie et al. (2) reported a prevalence of intellectual disability of somewhat <1%, but more recent studies have reported a rate of 1.2% in American children aged 3–17 years (3, 4). Although the prevalence of ID is not the highest among the neurodevelopmental disorders, ID is a chronic disorder that imposes a heavy burden on the family, and is among the top 20 most costly disorders (5, 6). The comorbidity or co-occurrence of mental disorders and neurological illness is common in children and adolescents with ID and affects both their clinical progression and the outcomes of interventions (5, 7–9). The most common co-occurrent mental problems in children are autistic spectrum disorders (ASD), attention-deficit/hyperactivity disorder (ADHD) and behavioral and emotional problems, which are significantly related to the development of different domains of adaptive behavior (5, 9–13). Independently of comorbid disorders, it has been estimated that there are several overlapping cognitive difficulties in ID related to attention (14–16), learning (15–18), memory (15, 18, 19), perceptive and visuospatial skills (17, 20, 21), executive functions (15, 18, 22), processing speed (22), and communication (15, 23–25).

In the field of disability management, functional and psychosocial interventions are used most frequently, but cognitive interventions have also yielded positive results (26–28). Cognitive training refers specifically to repeated practice in a specific domain to obtain both cognitive and behavioral improvement (29). Although there are few evidence-based strategies available, professionals tend to adapt materials to meet the needs of subjects with ID to overcome difficulties in their day-to-day lives (15, 30). Recently, the number of studies describing and evaluating skills training programs (31–33) has been on the rise, coinciding with the exponential development of information and communication technologies (ICT). Browing et al. (34) were pioneers in using computers to assess the effectiveness of community skills training in children with ID. Digital technologies have easy, clear objectives and instructions, and their virtual environment, striking colors, and entertaining music and sounds can make them attractive and useful tools for interventions with subjects with ID. Although the use of these technologies has increased in recent years with benefits reported in aspects like adaptive behaviors and learning, such as communication and socialization in small children with ID, research focusing on skills generalization and technology use is necessary (35). More specifically, virtual reality has been recommended as a means by which to practice or teach cognitive and emotional skills, robots have been suggested as a way to stimulate and engage children with ID, and handheld or multimedia devices have been recommended as learning supports. Digital media using interactive computer software (31, 32, 36–38) and web-based applications expressly designed to train and practice skills through smartphones or tablets (33, 39, 40) have both been used in subjects with ID. These programs have a fixed number of sessions of specific lengths, facilitating the process of recording performance measurements as well as longitudinal follow-up. In addition, these programs allow for both the provision of reward feedback and the adjustment of the difficulty of the task. For years, subjects with ID have been using technology to overcome their motor, communication and visual impairments (41), and these devices have contributed to facilitating their performance of day-to-day activities (42, 43). However, in order to fully take advantage of digital interventions (44–46), people with ID may need longer training periods and easier tasks to obtain the most benefit (47).

Due to the number and diversity of skills training programs available through digital devices for people with ID, it is important to describe which digital interventions and media have been developed, as well as which are the most effective. Programs and devices have been used to support language learning and communication (48, 49), daily living skills, time perception and imagination (42), executive function (50, 51), emotional skills (52) and to reduce behavioral problems (33). Due to the lack of systematic reviews conducted to assess the efficacy of digital interventions in children and adolescents with ID, our review focuses on this specific age group and encompasses all digital technology currently in common use. The aims of this study are (1) to assess the use of digital devices in children and adolescents with ID and the effects derived from their use on cognitive functions (e.g., attention, memory, executive functions and language), academic and behavioral skills, daily routines, and social skills, and (2) to determine whether this methodology can be considered a therapeutic tool for subjects with ID. This systematic review will contribute to bringing to light the hard work done with this specific population and will constitute a step forward for the inclusion of people with ID in society and for the improved quality of life for children and adolescents with ID by offering them modern, effective interventions.

## Materials and Methods

Prior to the literature search, we registered with the PROSPERO database (register number CRD42019121219) and created a detailed protocol in accordance with the Preferred Reporting Items for Systematic Review and Meta-Analysis Protocol (PRISMA-P) (53).

### Literature Search Strategy and Information Sources

A systematic literature search of SCOPUS, the Web of Science and PsycINFO was carried out that ended in February 2020. The following search formula was created with the following free terms: (“intellectual disability” OR “mental retardation” OR “neurodevel^*^ retardation” OR “cognitive disability”) AND (“self-help devices” OR “video games” OR “virtual reality” OR “APPS” OR “tablets” OR “Ipad” OR “computer*”) AND (“child” OR “adolescent”).

### Study Selection Process and Eligibility Criteria

The systematic review and the selected studies were organized according to the participants, interventions, comparators, outcome measures, and study design (PICOS). Participants were children and adolescents with ID (mild to profound) or with syndromes associated with ID. All studies included therapeutic interventions using digital devices such as virtual reality, computers (including laptops), touch screens, input devices and handheld devices [smartphones, personal digital assistants (PDA), tablets].

Outcome measures obtained using non-standardized or standardized tests were included if the dependent variables were related to either the cognitive, social, emotional or behavioral domains. The study designs included in our analysis were experimental studies, randomized and quasi-experimental (non-randomized or without control group). We included articles published in peer-reviewed journals in English or Spanish.

We excluded studies whose participants were parents or professionals, or had mental illness, traumatic brain injury or sensorial affection. In addition, we excluded studies that did not meet the previously defined PICOS characteristics or which contained poor empirical data. Case studies, reviews, abstracts and communications from scientific meetings and qualitative studies were not considered.

**Figure 1:**
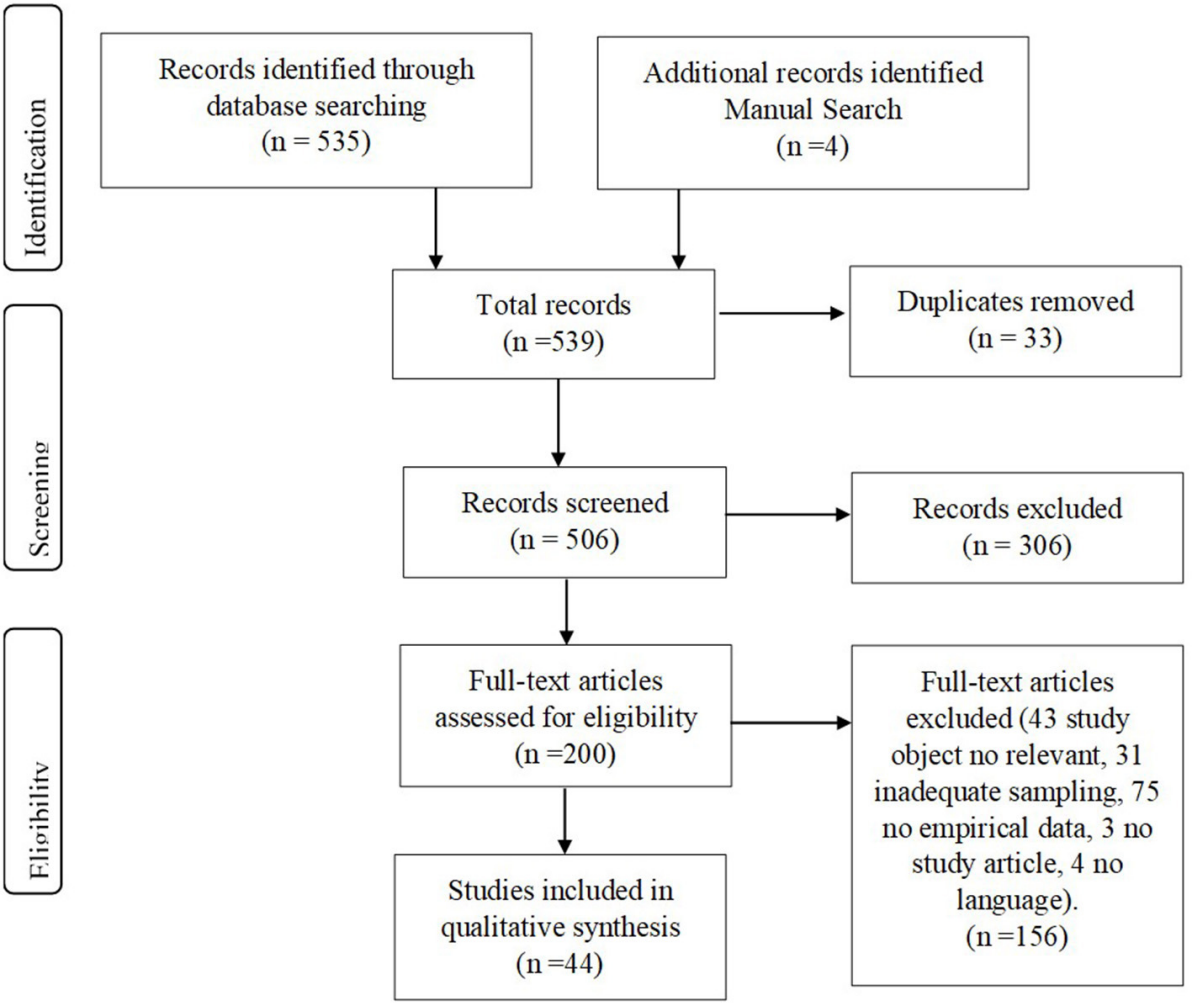
Flow chart for the article selection process.

The inclusion of the studies was independently reviewed by two authors. A form with inclusion criteria was designed and reviewed by all authors. In the first round, titles and abstracts of articles were selected in accordance with the form. In the second round, we assessed the full-text articles for their selection based on the inclusion criteria. In some cases, we requested the full text from the authors. Duplicated articles were removed. When necessary, any disagreement was discussed with a third author. We created a PRISMA flow chart to track the studies we included and discarded [(54); Figure 1].

### Risk of Bias Assessment

Three reviewers independently assessed the risk of bias for each study using the modified Cochrane Collaboration tool (55) for randomized controlled trials. Bias was assessed as a judgment (high, low or unclear) for individual elements from five domains (selection, performance, attrition, reporting, and other). We converted this score to a quality assessment, indicating that high risk of bias equals low quality, low risk of bias is equal to high quality and unclear risk of bias is equal to moderate quality. For non-randomized studies, we assessed the quality level using Newcastle-Ottawa Quality Assessment Scale (56). Bias was assessed as a judgement (good, low) for individual elements from three domains (selection, comparability and ascertainment), resulting in a total score. The categorization of the quality assessments is reflected in the summary tables.

## Results

### Study Characteristics

The flow chart included as Figure 1 illustrates the process of selection for the articles included in this systematic review. First, studies were identified in databases (*n* = 535) and manual searches (*n* = 4), and then duplicates were removed (*n* = 33). The titles and abstracts of the remaining 506 publications were screened to select articles that met the inclusion criteria, and 306 articles were excluded. The remaining 200 full texts were carefully examined. Then the articles that did not report an intervention or that were beyond the scope of this systematic review were excluded (*n* = 156). In the end, a total of 44 articles were included in this review. The general characteristics are summarized in Table 1, while detailed information for each study is summarized in Tables 2–**4**.

**Table 1:** Study characteristics according to psychological outcomes.

**Year of publication**	**Executive functions (*n =* 10)**	**Basic cognition skills (*n =* 18)**	**Academic skills (*n =* 7)**	**Behavioral and social skills (*n =* 22)**	**Total**
≤ 1999	0	6	1	4	11
2000–2009	1	3	0	1	5
2010–2020	9	9	6	17	41
**Origin**					
Europe	7	6	4	11	28
Americas	0	6	2	3	11
Oceania	1	3	1	3	8
Asia	2	3	0	5	10
**Number ID participants**					
0–49	5	12	4	14	35
50–99	5	6	3	8	22
**Design**					
Randomized	8	11	5	12	36
Quasi-experimental non-randomized	1	5	0	4	10
Quasi-experimental without control group	1	2	2	6	11
**Technology used**					
PC	9	12	5	16	42
Handheld	1	6	2	7	16
Ids	1	0	3	3	7
Touch screen	1	1	0	0	2
NR	0	0	0	1	1
**Evaluated functions**					
Total	15	20	7	25	67
**Tools**					
Standardized tests	9	9	2	12	32
Non-standardized quantitative measures	6	11	5	13	35

**Table 2:** Reviewed studies focusing on executive functions.

**References**	**Origin**	**Sample size, type, age**	**Design**	**Technology (task)**	**Duration (post-test time/follow-up time)**	**Evaluated functions**	**Tools**	**Outcomes**	**Quality article**
Bennett et al. (46)	UK	*N =* 25, ID, age: 7–12	Randomized. EG: intervention CG: usual treatment	PC (repeated sequences)	3 × 25 min/wk for 10–12 wks (2 wks/4 months)	Working memory	BRIEF, AWMA, NQM (Cogmed tasks)	EG performed significantly for general EF and working memory and maintained to follow-up.	Low
Bruttin (62)	Switzerland	*N =* 36, TD, *N =* 26, ID, age: 4–18	Randomized. EG1:ID, EG2: TD	PC+ touch screen (complete matrices inferring relations)	16 trials × 2 sessions	Reasoning	NQM (monitored performance)	EG1 performed higher with external memories in task.	Low
Delavarian et al. (60)	Iran	*N =* 12, ID, age: 9–14	Quasi-experimental non-randomized. EG: training program CG: school-routine	PC (repeated sequences, identification similarities)	5 × 30 min/wk for 4 wks (EP/1 wk)	Working memory	WISC-IV (numerical forward, backward subtests) NQM (dual tasks)	EG performed significantly higher than CG. Task performance improved significantly in EG in visual tasks in post-test and follow-up.	Low
Glaser et al. (61)	France	*N =* 10, ID, age: 7–10	Quasi-experimental without CG	PC (repeated sequences, matching cards)	4 × 20 min/wk for 12 wks (EP/6 months)	Working memory	WISC-IV (digit span, letter-number sequence, arithmetic subtests), CMS (sequences, picture location subtests)	Task performance improved significantly in whole group.	Low
Jansen et al. (63)	Netherlands	*N =* 58, ID, age: 12–15	Randomized. EG:PC training, CG: usual treatment	PC (arithmetic operations)	4 × NR min/wk for 5 wks (2 months/NF)	Working memory	NQM (memory and spatial span)	No significant changes.	Low
						Inhibition	Stroop task	No significant changes.	
Kirk et al. (50)	Australia	*N =* 76, ID, age: 4–11	Randomized. EG: software program, CG:B: non-adaptive software	Handheld (identification, discrimination and inhibition tasks).	5 × 20 min/wk for 5 wks (5–6 wks/3 months)	Working memory	BRIEF, WMRS	Both groups improved in general EF and working memory, but no significant differences between groups in post-test or follow-up.	Low
Ottersen and Grill (47)	Norway	*N =* 21, ID, age: 8–13	Randomized. EG1: long adaptive training, EG2: short adaptive training Söderqvist study (45) CG: non-adaptive training Söderqvist study (45)	PC (repeated sequences)	5 sessions × 10–23 wks (NR/NF)	Working memory	AWMA (odd-one-out subtest) NQM (word span)	EG1 performed significantly higher than CG in visuospatial working memory. EG1 improved more than EGD.	Low
				PC (classification, sequential logical order, repeated patterns)		Reasoning	WPPSI-III (block design, matrix reasoning, word reasoning subtests)	EG1 and EG2 performed significantly higher than CG in non-verbal reasoning. EG1 performed significantly better than EG2 in nonverbal reasoning.	
Passig (59)	Israel	*N =* 87, ID, age: 9–21	Randomized. EG: 3D IVR training, CG1: 2D pictorial training, CG2: no training	PC+ Ids (logical time sequence)	2 × 20 min/wk for 1 month (NR/NF)	Planning	KABC-II (pictures series subtest) NQM (logical)	Task performance improved significantly in EG and CG1. EG performed slightly better than CG.	Low
Söderqvist et al. (45)	Norway	*N =* 52, ID, age: 6–12	Randomized. EG: adaptive training, CG: non-adaptive training	PC (repeated sequences).	5 × 20 min/wk for 5 wks (EP/1 year)	Working memory	AWMA (odd one out subtest) NQM (memory span)	Task performance improved in EG in post-test but not in follow-up.	Low
				PC (classification, sequential logical order, repeated patterns)		Reasoning	WPPSI (block design subtest), CPM	Task performance improved in EG in post-test but not in follow-up.	
Van der Molen et al. (64)^*^	Netherlands	*N =* 95, ID, age: 13–16	Randomized. EG1: adaptive training EG2: non-adaptive training CG: control training	PC (identify and recall differences between figures)	3 × 6 min/wk for 5 wks (EP/10 wks)	Working memory	NQM (memory and visual span tasks)	All groups performed significantly better in verbal working memory. Maintained in follow-up.	Moderate
						Inhibition	Stroop task	No significant changes observed in post-test or follow-up.	
						Reasoning	SPM	No significant changes observed in post-test or follow-up.	

* *Article assesses various functions. Sample: ID, intellectual disability. Design: EG, experimental group; CG, control group. Technology: Ids, input devices; PC, Computer. Duration: EP, at the end of the program; NF, no follow-up; NR, not reported; WK, week. Evaluated Functions: EF, executive functions. Tools: AWMA, Automated Working Memory Assessment; BRIEF, Behavior Rating Inventory of Executive Function; CMS, Children's Memory Scale; CPM, Raven's Colored Progressive Matrices; KABC, Kaufman Assessment Battery for Children; NQM, Non-standardized quantitative measures; SPM, Raven Standard Progressive Matrices; WISC, Wechsler Intelligence Scale for Children; WMRS, Working Memory Raging Scale; WPPSI, Wechsler Preschool and Primary Scale of Intelligence*.

### Executive Functions

As detailed in Table 2, 10 studies assessed the effect of interventions on executive functions, some of them also evaluated reasoning. Following theoretical models from different authors and the methodology of some of the studies (57, 58) reasoning has been included in executive function analyses. Through all the studies, executive functions were assessed in 462 children and adolescents with ID. The majority of these studies were published within the last decade and only one [10%] was published between 2000 and 2009 (59). Sample sizes were small in all of the studies we analyzed (range *n* = 10 to *n* = 95). Participant age ranged from 4 to 21. Most of the studies used randomized designs, except two [20%]: a quasi-experimental non-randomized study (60), and a quasi-experimental study without control group design (61). Most of the interventions used personal computers (*n* = 9) [75%] (45–47, 59–64). There was great variability in the tasks used: repeated sequences (45–47, 60, 61), matrices (62), identification and discrimination (50, 60, 64), classification and ordering (45, 47, 59) repeated patterns, mathing and arithmetic operations (45, 61, 63). Session interventions generally last for a period of between 20 and 30 min or until the completion of a concrete number of tasks or sessions (47, 62). The duration of the interventions ranged from 4 to 23 weeks. Post-test evaluations were generally performed only at the end of the program (45, 60, 61, 64) or at 1 or 2 months (50, 63). Almost half of the studies did not refer to any follow-up monitoring, and the interval of the rest was between 2 months to 1 year (45, 46, 50, 61, 64). The most common function evaluated was working memory (45–47, 50, 60, 61, 63, 64) and reasoning (45, 47, 62, 64). Outcomes measures were obtained by means of behavior tests that assess executive functions such as Behavior Rating Inventory of Executive Function BRIEF and Automated Working Memory Assessment AWMA (45–47, 50), neuropsychological tests applied to children as working memory subtests of Weschler Intelligence Scale (45, 47, 60, 61) and Stroop Task (63, 64). Nine [60%] studies used standardized tests and six [40%] studies used non-standardized quantitative measures. Of the studies analyzed, six [60%] reported significant improvements and the remainder reported some improvements or non-significant changes (45, 50, 62–64). Finally, our assessment of the quality of the studies determined that nine [90%] were low quality and one [10%] was moderate (64).

### Basic Cognition Skills

Eighteen studies assessed the effect of interventions on basic cognition skills as their main focus (Table 3). A total of 592 subjects with ID were evaluated in basic cognition skills. Nine [50%] of the studies were published in the past decade, six [33%] were published before 1999 and three [16%] between 2000 and 2009. Sample sizes were usually small (range *n* = 4 to *n* = 95). Subject age ranged between 1 and 22. Eleven [61%] studies had a randomized design, the rest were quasi-experimental non-randomized (65–69) and quasi-experimental without control group (70, 71). In terms of the technology used, 12 [63%] of the studies used personal computer devices for their interventions (45, 64–75). Language was the most common function evaluated with 14 [70%] of the studies analyzed (45, 50, 64–72, 74, 76, 77) followed by attention, which were the focus of three [15%] of the studies analyzed (45, 75, 78). There was great heterogeneity regarding the tasks used, with the most common related to concept matching (65, 71, 72, 77), sentence construction, pronunciation, drawing, writing, answering questions (51, 66–70, 72, 74, 76) identification and discrimination tasks (50, 51, 64, 67, 78). In general, the duration of the sessions was between 20 and 30 min. In some studies, the length of the session was adjusted to the completion of the tests (72, 77, 79). Fourteen [77%] of the studies analyzed had a duration of between 1 and 4 months. Eight [44%] of the studies analyzed did not specify when post-test assessments were administered. When this data was available the range varied between immediately after the program and 6 weeks. Regarding follow-up, only a few studies assessed long-term outcomes of between 1 month and 1 year (45, 50, 64, 66, 74, 75, 78). A great number of studies, 11 [55%] to be exact, used non-standardized quantitative measures, the remainder (*n* = 9) [45%], used standardized tests. Of the studies analyzed, 10 [55%] reported significant benefits [all in language (*n* = 7)], four [22%] obtained non-significant improvements, and in the remaining, improvements were similar between groups or no improvement was observed (51, 68, 74). Fifteen [83%] studies were categorized as low quality, two [11%] as having moderate quality and one [5%] as high quality.

**Table 3:** Reviewed studies focusing on basic cognitive skills.

**References**	**Origin**	**Sample size, type, age**	**Design**	**Technology (task)**	**Duration** **(post-test time/ follow-up time)**	**Evaluated functions**	**Tools**	**Outcomes**	**Quality article**
Alcalde et al. (72)	Spain	*N =* 60, ID, age: 8–16	Randomized. EG: PC training, CG: drill-and-practice	PC+ touch screen (response questions, match opposite concepts)	12 trials × 4 sessions (NR/NF)	Language (vocabulary)	BCAT	EG performed significantly better than CG.	Low
Conners and Detterman (65)	US	*N =* 37, ID, age: 9–22	Quasi-experimental non-randomized. EG: PC training, CG: usual treatment.	PC (match audio word with visual word)	3 × 10 min/wk for 4–10 wks (NR/NF)	Language (vocabulary)	NQM (simple learning tasks)	Task performance improved significantly in EG in short time.	Low
Coutinho et al. (51)	Canada	*N =* 20, special needs, age: 4–7	Randomized. EG: handheld training, CG: traditional occupational therapy.	Handheld (visual discrimination, tracing, mazes)	2 × 40 min/wk for 10 wks (1 wk after end/NF)	Visual-motor integration	Beery VMI, M-FUN (visual motor subtest)	Task performance improved in 2 groups. EG group performed similarly to CG.	Low
Felix et al. (76)	Mexico	*N =* 12, ID, age: 6–15	Randomized. EG: handheld training, CG: conventional training	Handheld (repeat pronunciation, draw words, letters, and figures)	5 × 60 min/wk for 16 wks (NR/NF)	Language (literacy)	NQM (literacy and letter identification tasks)	EG1 performance improved significantly more than CG.	Low
Fujisawa et al. (77)	Japan	*N =* 16, ID, age: 11–18	Randomized. EG1, EG2: A-B animated pictos, C-D static pictos. EG3, EG4: A-B static pictos, C-D animated pictos	Handheld (match action word with static and animated symbol)	8 trials × 2 sessions (1 wk after end/NF)	Language (vocabulary)	NQM (naming tasks)	Animated pictograms help to learn vocabulary better than static pictograms.	Low
Gillette and Depompei (79)	US	*N =* 15, TBI, *N =* 20, ID, age: 6–20	Randomized. EG1: list, EG2: planner, EG3: PDA, EG4: PDA	Handheld (complete daily schedule tasks on time)	8 tasks for 8 wks (NR/NF)	Temporal orientation	NQM (monitored performance)	EG3 and EG4 performed significantly better than EG1 and EG2.	Low
Heimann et al. (66)	Sweden	*N =* 21, ASD/TD, *N =* 9, ID, age: 2–13	Quasi-experimental non-randomized	PC (learn vocabulary, create sentences, create sentences after watch animation)	1 × 21–32 min/wk for 3–4 months (last wk of intervention/6 months)	Language (literacy)	NQM (reading, communication, phonological awareness tasks)	Task performance improved significantly in 3 groups in post-test. In follow-up, improved phonological but not reading.	High
Herring et al. (70)	UK	*N =* 8, ID, age: 7–19	Quasi-experimental without CG	PC (repeat sounded letter, mix letter sounds to read a word)	1–2 × NR min/wk for 13–18 wks (NR/NF)	Language (literacy)	DIBELS-VI (ISF, PSF, NWF, WUF subtests), WRAPS	Task performance improved in whole group.	Low
Kirk et al. (78)	Australia	*N =* 76, ID, age: 4–11	Randomized. EG: attention training, CG: non-adaptive training	Handheld (identification, discrimination and inhibition tasks)	5 × 20 min/wk for 5 wks (EP/2 months)	Attention	WATT	Task performance improved significantly in EG in selective attention in post-test and follow-up.	Low
Kirk et al. (50)^*^	Australia	*N =* 76, ID, age: 4–11	Randomized. EG: software program, CG:B: non-adaptive software	Handheld (identification, discrimination and inhibition tasks)	5 × 20 min/wk for 5 wks (5–6 wks/3 months)	Language (literacy)	PPVT-4, PAT	Both groups improved, but no significant differences between groups in post-test or follow-up.	Low
Margalit and Roth (67)	Israel	*N =* 18 LD, *N =* 18, ID, age: 11–16	Quasi-experimental non-randomized. EG1:LD, EG2: ID	PC (identification of letters on keyboard, games, word and sentences exercices, typing)	2 × 45 min/wk for 3 months (NR/NF)	Language (literacy)	NQM (spelling tasks)	Task performance improved significantly in EG2.	Low
Oconnor and Schery (74)	US	*N =* 8, ID, age: 1–2	Randomized. EG1: PC-aided intervention+ traditional therapy. EG2: traditional therapy+ PC aided-intervention	PC (pronounce the object appearing on the screen)	2 × 20 min/wk for 6–10 wks (1 wk after end/1 month)	Language (vocabulary)	PPVT-R, PEAL, ICP, VABS	In post-test, task performance improved in 2 groups, EG performed similar to CG, parents reported progress in communication. New vocabulary maintained in follow-up.	Low
Rezaiyan et al. (75)	Iran	*N =* 60, ID, age: children	Randomized. EG: PC training. CG: no training	PC (mazes)	35 × 20–30 min (EP/5 wks)	Attention	T-PS	EG performed significantly higher than CG in post-test. EG performed similar to CG in follow-up. EG performance improved significantly post-test and follow-up.	Low
Söderqvist et al. (45)^*^	Norway	*N =* 52, ID, age: 6–12	Randomized. EG: adaptive training, CG: non-adaptive training	PC (repeated sequences, classification, sequential logical order, repeated patterns)	5 × 20 min/wk for 5 wks (EP/1 year)	Attention	NEPSY-II (auditory attention subtest)	Task performance improved in EG in sustained attention post-test but not in follow-up.	Low
						Language	NEPSY-II (instructions subtest)	Task performance improved in EG in post-test but not in follow-up.	
Tjus et al. (68)	Sweden	*N =* 50, ASD/CP/LD/ ADHD, *N =* 11, ID, age: 9–17	Quasi-experimental non-randomized	PC (create sentences)	NR frequency and duration session. 2–4 months (NR/NR)	Language (literacy)	NQM (reading tasks)	Task performance not improved in ID group.	Moderate
Vacc (69)	US	*N =* 4, ID, age: 13–14	Quasi-experimental non-randomized. EG1: ABAB, EG2: BABA, (A: handwriting, B:PC)	PC (complete letters by handwriting and computer)	6 × 45 min (NR/NF)	Language (literacy)	NQM (writing tasks)	Task performance improved in whole group.	Low
Van Bysterveldt et al. (71)	New Zealand	*N =* 10, ID, age: 4–5	Quasi-experimental without CG	PC (match phonemes, letter name-sound-phoneme)	2 × 20 min/wk for 18 wks (EP/NF)	Language (literacy)	PPVT-III, PLS-4, HAPP-3. NQM (letter knowledge, phonological awareness tasks)	Task performed improved significantly in whole group.	Low
Van der Molen et al. (64)^*^	Netherlands	*N =* 95, ID, age: 13–16	Randomized. EG1: adaptive training EG2: non-adaptive training CG: control training	PC (identify and recall differences between figures)	3 × 6 min/wk for 5 wks (EP/10 wks)	Short-term memory	NQM (memory and visual span tasks)	Task performance improved significantly in EG1 and EG2 in post-test, maintained in follow-up.	Moderate
						Language (literacy)	NQM (reading and comprehension tasks)	EG1 and EG2 performed significantly better in comprehension post-test. Maintained in follow-up.	

* *Article assesses various functions. Sample: ADHD, attention deficit hyperactive disorder; ASD, autism spectrum disorder; CP, cerebral palsy; ID, intellectual disability; LD, learning disabilities; TBI, traumatic brain injury; TD, typical development. Design: EG, experimental group; CG, control group; PDA, personal digital assistant. Technology: PC, computer. Duration: EP, at the end of the program; NF, no follow-up; NR, not reported; WK, week. Tools: BCAT, Basic Concepts Assessment Tests; BEERY VMI, Bucktenica Developmental Test of Visual-Motor Integration; DIBELS, Dynamic Indicators of Basic Early Literacy Skills; HAPP, Hodson Assessment of Phonological Patterns; ICP, Initial Communication Processes Observational Scales; ISF, Initial Sound Fluency; M-FUN, Miller Function & Participation Scales; NEPSY, Developmental Neuropsychological Assessment; NQM, non-standardized quantitative measures; NWF, Nonsense Word Fluency; PAT, Phonological Abilities Test; PEAL, Programs for Early Acquisition of Language; PLS, Pre-School Language Scale; PSF, Phonemic Segmentation Fluency; PPVT, Peabody Picture Vocabulary Test; T-PS, Touluse-Pieron Scale; VABS, Vineland Adaptive Behavior Scale; WATT, Wilding Attention Battery; WRAPS, Word Recognition and Phonic Skills; WUF, Word Use Fluency*.

### Academic Skills

As detailed in Table 4, seven studies assessed the effect of interventions on academic skills adding a total of 264 subjects evaluated. Most of them, concretely six [86%] studies were published between 2010 and 2020 and one study [14%] was published before 1999. Sample sizes were small in all studies analyzed (range *n* = 3 to *n* = 95). Participant age ranged between 3 and 23 years. Regarding design of the studies, most of them (*n* = 5) [71%] were randomized design and two [28%] were quasi-experimental without control group (80, 81). Respecting technology used in the interventions, half of the studies (*n* = 5) [50%] used personal computer (63, 64, 73, 80, 82), some of them also used input devices (*n* = 3) [30%]; and the remaining two [20%], used handheld (78, 81). Concerning evaluated functions, mathematics was the most common (*n* = 6) [85%] (50, 63, 64, 80–82). Regarding the tasks used, existed a great heterogeneity. The most common were related to arithmetic operations (63, 80), matching or response questions (81, 82) and identification and classification (50, 64, 73). In general, the duration of the sessions was between 10 and 30 min. Most of the studies (*n* = 6) [85%] had a duration ranged between 1 and 4 months. Four [57%] studies specify post-test assessments, from just at the end of the intervention to 2 months. Only three [42%] studies conducted long-term assessment, they were ranged between 10 weeks and 3 months (50, 64, 80). In reference to assessment tools, the majority of the studies analyzed used non-standardized quantitative measures (*n* = 5) [71%], and the rest (*n* = 2) [28%], used standardized tests. The outcomes reported from the studies analyzed, two [29%] showed significant improvements (64, 82) and the rest (*n* = 5) [71%] reported some improvements or non-significant changes. Six [85%] studies were categorized as low quality and one [15%], as moderate quality.

**Table 4:** Reviewed studies focusing on academic skills.

**References**	**Origin**	**Sample size, type, age**	**Design**	**Technology (task)**	**Duration** **(post-test time/follow-up time)**	**Evaluated functions**	**Tools**	**Outcomes**	**Quality article**
Cress and French (73)	US	*N =* 58, Adults/TD, *N =* 15, ID, age: 3–9	Randomized: EG1: touchscreen, EG2: mouse, EG3: keyboard, EG4, trackball, EG5: locking trackball	PC+ Ids (displacement, classification).	NR frequency. 30 min for 2–4 weeks (NR/NF)	Computer learning	NQM	Task performance worse in trackball and locking trackball. Task performance improved with mouse.	Low
Hammond et al. (82)	US	*N =* 11, FXS, *N =* 11, ID, age: 10–23	Randomized. EG1: FXS, EG2: idiopathic ID	PC + Ids (match fractions-pie charts-decimals)	15 min × 2 days (NR/NF)	Mathematics	NQM (math tasks)	Task performed improved significantly in 2 groups. EG2 performed higher than EG1.	Low
Jansen et al. (63)^*^	Netherlands	*N =* 58, ID, age: 12–15	Randomized. EG1: PC training, CG: usual treatment	PC (arithmetic operations)	4 × NR min/wk for 5 wks (2 months/NF)	Mathematics	TTA	Math task performance similar in EG1 and CG.	Low
Kirk et al. (50)^*^	Australia	*N =* 76, ID, age: 4–11	Randomized. EG: software program, CG: non-adaptive software	Handheld (identification, discrimination and inhibition tasks)	5 × 20 min/wk for 5 wks (5–6 wks/3 months)	Mathematics	GAN, TEMA-3	EG not improved in math in post-test, but yes in follow-up. No differences between groups in cardinality.	Low
Stasolla et al. (80)	Italy	*N =* 6, CP, age: 9–12	Quasi-experimental without CG.	PC+ Ids (arithmetic operations, writing, geography)	5 × 20 min/wk for 6 wks (EP/3 months)	Mathematics, general knowledge	NQM (monitored performance)	Task performance improved in whole group in post-test and follow-up. Combined interventions were better than single.	Low
Stasolla et al. (81)	Italy	*N =* 3, ASD + ID, age: 8–10	Quasi-experimental without CG	Handheld (response questions about literacy, arithmetic operations, history, geography, natural sciences)	20 × 10 min/wk for 4 months (NR/NF)	Mathematics, general knowledge	NQM (monitored performance)	Task performance improved in whole group.	Low
Van der Molen et al. (64)^*^	Netherlands	*N =* 95, ID, age: 13–16	Randomized. EG1: adaptive training EG2: non-adaptive training CG: control training	PC (identify and recall differences between figures)	3 × 6 min/wk for 5 wks (EP/10 wks)	Mathematics	NQM (arithmetic tasks)	EG1 and EG2 performed significantly better in post-test. Maintained in follow-up.	Moderate

* *Article assesses various functions. Sample: ASD, autism spectrum disorder; CP, cerebral palsy; ID, intellectual disability; FXS, X-Fragile syndrome; TD, typical development. Design: EG, experimental group; CG, control group. Technology: Ids, input devices; PC, computer. Duration: EP, at the end of the program; NF, no follow-up; NR, not reported; WK, week. Tools: GAN, Give-a-number; NQM, non-standardized quantitative measures; TEMA, Test of Early Mathematics Ability; TTA, TempoTest Automatiseren*.

### Behavioral and Social Skills

The studies that assessed the effect of interventions on behavioral and social skills are described in Table 5. Through all the studies, 759 children and adolescent with ID were assessed. Again, most of these studies were published in the last decade (*n* = 17) [77%]. Sample sizes were mostly small (range *n* = 3 to *n* = 87) and the age ranges were between 4 and 31 years old. In terms of the design of the studies analyzed, 12 [54%] had a randomized design, six [27%] a quasi-experimental design without CG (34, 61, 80, 81, 83, 84) and four [18%] a quasi-experimental non-randomized design (85–88). The most common devices used were personal computers (34, 45, 61, 80, 83–85, 87–94) and handheld devices (50, 52, 78, 81, 84, 94, 95). The most commonly evaluated functions were behavioral (45, 50, 52, 78, 80, 81, 86–88, 90, 92) and social skills (34, 61, 83–85, 87, 93–95). The most common tasks proposed were matching (45, 52, 61, 83, 85, 87, 94), combination with real life tasks (52, 84, 89, 92, 95) and sequences (45, 83, 88, 90). Sessions had a general duration of between 20–30 min and 55–90 min. Generally, the duration of the interventions was between 2 and 4 months. Time to post-test was absent in nine [40%] the studies, only at the end of the program in eight [36%] studies, and between 1 week and 3 months post program in five [22%]. Most of these studies did not include any follow-up. Non-standardized quantitative measures (*n* = 13) [52%] and standardized tests (*n* = 12) [48%] were used about equally. The outcomes of the interventions reported indicate that almost half resulted in significant benefits (*n* = 10) [45%], and the remainder obtained no significant improvements (*n* = 11) [50%], or non-significant changes (*n* = 1) [5%]. Of the significant benefits reported, nine [50%] studies correspond to social skills and three [30%] to behavioral skills. Seventeen [77%] of the studies were categorized as low quality, four [18%] as moderate and one [5%] as high.

**Table 5:** Reviewed studies focusing on behavioral and social skills.

**Reference**	**Origin**	**Sample size, type, age**	**Design**	**Technology (task)**	**Duration** **(post-test time/follow-up time)**	**Evaluated functions**	**Tools**	**Outcomes**	**Quality article**
Browning et al. (34) (Study 1)	US	*N =* 26, ID, age: 14–31	Randomized. EG1: high school students, EG2: high school students, EG3: adults	PC (response questions)	5 × 55 min/wk for 2 wks (NR/NF)	Social (community skills)	SPIB (budgeting subtest)	Task performed improved significantly in both groups.	Low
Browning et al. (34) (Study 3)	US	*N =* 36, LD, *N =* 78, ID, age: 13–21	Quasi-experimental without CG	PC (response questions)	5 × 55 min/wk for 2 wks (NR/NF)	Social (community skills)	K-BT, CAT	Task performance improved significantly in all groups.	Moderate
Choi et al. (89)	China	*N =* 29, ID, age: 6–11	Randomized. EG: PC training. CG: conventional training	PC + Ids (washing hands step by step associated with a game)	2 × 30 min/wk for 2 months (NR/NF)	ADL (hand washing)	NQM (hand washing checklist)	EG performed slightly higher than CG.	Low
Eden and Bezer (90)	Israel	*N =* 87, ID, age: 9–21	Randomized. EG: 3D IVR training, CG: 2D pictorial training	PC+ Ids (sort sequence)	2 × 20 min/wk for 1 month (EP/NF)	Behavior (adaptive)	NQM (observation checklist)	Self-sufficiency increased in 2 groups. EG performed more self-sufficiently than CG. Moderate ID performed with slightly higher mediation than mild ID. EG displayed higher concentration and less stress.	Low
Fage et al. (95)	France	*N =* 5, ASD, *N =* 5, ID, age: 13–17	Randomized. EG1: ASD trained group. EG2: ID trained group	Handheld (follow photo steps to accomplish a task)	60 min/wk for 3 months (NR/NF)	Social (communication)	NQM (observation checklist)	Task performed improved significantly in EG2. EG2 needed longer intervention.	Low
Fage et al. (52)	France	*N =* 29, ASD, *N =* 19, ID, age: 12–17	Randomized. EG1: ASD + app, CG: ASD, EG2: ID + app.	Handheld (identify their emotion with emoticon, practice auto-regulation strategy)	1 × 60 min/wk for 3 months (1 wk after the end/NF)	Behavior (adaptive)	SRS, EQCA-VS	EG2 self-regulation behaviors lower than EG1.	Low
						Emotion (self-regulation)	EWFT, Self-LEAS-C	Apps groups improved in post-test. EG1 performed significantly higher than CG and EG2.	
Fatikhova and Saifutdiyarova (85)	Russia	*N =* 40, ID, age: 8–9	Quasi-experimental non-randomized. EG: PC intervention. CG: non-PC intervention	PC (match emotion with portrait and scene picture)	NR (NR/NF)	Social (emotional intelligence)	NQM (monitoring performance)	EG performed higher in social recognition than CG.	Moderate
Glaser et al. (61)^*^	France	*N =* 10, ID, age: 7–10	Quasi-experimental without CG	PC (facial puzzles, match emotion/eyes/facial expression/emotion/ name/situation)	4 × 20 min/wk for 12 wks (EP/6 months)	Social (emotional intelligence)	BFRT, CPM, NQM (monitored performance)	Task performance improved in post-test in whole group and maintained in follow-up.	Low
Grewal et al. (91)	India	*N =* 60, ID, age: NR	Randomized. EG: PC training, CG: conventional training	PC (watch digital information)	NR duration sessions and frequency for 9 months (3 months interval/NF)	ADL (oral hygiene)	F&H. NQM (observation checklist)	EG performed significantly higher than CG.	Low
Hetzroni and Banin (83)	Israel	*N =* 5, ID, age: 11–15	Quasi-experimental without CG	PC (identify adequate/non-adequate social behaviors related to watched video, sequences of behaviors, link behavior-consequences)	NR frequency and duration intervention. Session length 10–20 min (EP/1 month)	Social (community skills)	NQM (observation checklist)	Task performance improved in whole group.	Low
Kiewik et al. (86)^*^	Netherlands	*N =* 73, ID, age: 12–16	Quasi-experimental non-randomized. EG: e-learning intervention, CG: standard curriculum	NR (games, videos, quizzes, tests)	NR frequency and duration sessions. For 2 wks. (1 wk after end/NF)	Behavior (drug use)	NQM (questionnaire)	EG performed significantly worse in social pressure than CG.	High
Kirk et al. (78)^*^	Australia	*N =* 76, ID, age: 4–11	Randomized. EG: attention training, CG: non-adaptive training	Handheld (identification, discrimination and inhibition tasks)	5 × 20 min/wk for 5 wks (EP/2 months)	Behavior (non-adaptive)	SWAN	Lower symptomatology rated in post-test and follow-up.	Low
Kirk et al. (50)^*^	Australia	*N =* 76, ID, age: 4–11	Randomized. EG: software program, CG:B: non-adaptive software	Handheld (identification, discrimination and inhibition tasks)	5 × 20 min/wk for 5 wks (5–6 wks/3 months)	Behavior (non-adaptive)	DBC-P	Behavioral problems decreased in both groups. No significant differences between groups.	Low
Margalit et al. (87)	Israel	*N =* 73, ID, age: 11–15	Quasi-experimental non-randomized. EG: PC training, B: standard curriculum	PC (select solutions to conflictive situations)	2 × 20–25 min/wk for 3 months (NR/NF)	Social (emotional intelligence)	LQ, PR, SSRS	EG performed slightly better in social skills and socially accepted than CG. No significant changes in loneliness.	Moderate
						Behavior (non-adaptive)	ABS	EG reduced significantly disruptive behavior.	
Plienis and Romanczyk (88)	US	*N =* 13, ASD/ED/P, *N =* 4, ID, age: 4–14	Quasi-experimental non-randomized. EG1: AB, EG2: BA, (A: Adult-Instruction, B: PC-Instruction)	PC (sequences)	20 × 26 min (NR/NF)	Behavior (non-adaptive)	NQM (observational and monitored performance)	EG performed similarly in task as CG. EG improved in disruptive behavior.	Low
Raghavendra et al. (84)	Australia	*N =* 9 ID, age: 10–21	Quasi-experimental without CG	PC/Handheld (follow strategies to use computers)	1 × 75 min/wk for 3–4 months (EP/NF)	Social (communication)	COPM, GAS, NQM (observation checklist)	Task social media improved and communication with partners increased in whole group.	Moderate
Schuurmans et al. (92)	Netherlands	*N =* 19, TD, *N =* 18, ID, age: 11–16	Randomized, EG: PC training CG: no training	PC (practice relaxation technique)	2 × 30 min/wk for 4 wks (EP/4 months)	Emotion (symptoms)	SCAS- self report SCAS-P	EG significantly reduced anxiety in post-test, not in follow-up.	Low
						Behavior (non-adaptive)	SDQ- self report SDQ-P	EG significantly reduced externalizing behaviors problems in post-test, not in follow-up.	
Söderqvist et al. (45)^*^	Norway	*N =* 52, ID, age: 6–12	Randomized. EG: adaptive training, CG: non-adaptive training	PC (repeated sequences, classification, sequential logical order, repeated patterns,)	5 × 20 min/wk for 5 wks (EP/1 year)	Behavior (non-adaptive)	SDQ-P	No significant changes in behavior.	Low
Stasolla et al. (80)^*^	Italy	*N =* 6, CP, age: 9–12	Quasi-experimental without CG	PC+ Ids (arithmetic operations, write, geography)	5 × 20 min/wk for 6 wks (EP/3 months)	Behavior (adaptive)	NQM (monitored observation)	Positive participation increased in whole group.	Low
Stasolla et al. (81)^*^	Italy	*N =* 3, ASD+ID, age: 8–10	Quasi-experimental without CG	Handheld (response questions, arithmetic operations, history, geography, natural sciences).	20 × 10 min/wk for 4 months (NR/NF)	Behavior (non-adaptive)	NQM (monitored observation)	Task-focused behavior increased and stereotypic behavior reduced in whole group.	Low
Tjus et al. (93)	Sweden	*N =* 11, ASD, *N =* 9, ID, age: mean 11	Randomized. EG1:ID, EG2: ASD	PC (not reported)	NR frequency and duration session. 3–4 months (NR/NR)	Social (communication)	NQM (observation checklist)	Verbal expression improved significantly in whole group. EG1 no significant changes.	Low
Vasilevska and Trajkovski, (94)	Macedonia	*N =* 14, ASD, *N =* 20, ASD+ID, age: 7–15	Randomized. EG: PC training, CG: usual treatment	Handheld/PC (match emotion/facial expression/facial features/emoticon/situation, pairs of cards facial expression)	90 min/wk for 8 wks (1 wk after end/NF)	Social (emotional intelligence)	ECT	EG emotion cognition preformed significantly better than CG.	Low

* *Article assesses various functions. Sample: ASD, autism spectrum disorder; CP, cerebral palsy; ID, intellectual disability; ED, emotionally disturbed; LD, learning disabilities; P, psychosis; TD, typical development. Design: EG, experimental group; CG, control group; IVR, immersive virtual reality; 2D, two-dimensional; 3D, three-dimensional. Technology: Ids, input devices; NR, not reported; PC, computer. Duration: EP, at the end of the program; NF, no follow-up; NR, not reported; WK, week. Evaluated Functions: ADL, Activities of Daily Living. Tools: ABS, Aggressive Behavior Scale; BFRT, Benton-Face Recognition Test; CAT, Curriculum Application Test; CDI, Children's Depression Inventory; DBC-P, Developmental Behavior Checklist-Parent; COPM, Canadian Occupational Performance Measure; ECT, Emotion Comprehension Test; CPM, Raven's Colored Progressive Matrices; EQCA-VS, Quebec adaptive behavior scale for school; EWFT, Emotional Words Fluency Test; F&H, Frankl & Houpt behavior rating scale; GAS, Goal Attainment Scaling; K-BT, Knowledge-Based Test; LQ, Loneliness Questionnaire; NQM, Non-standardized Quantitative Measures; PR, Peer Rating; R-CMAS, Revised Children's Manifest Anxiety Scales; SCAS-P, Spence Children's Anxiety Scale-Parents Version; SDQ-P, Strengths and Difficulties Questionnaire-Parents Version; Self-LEAS-C, Self-Levels of Emotional Awareness Subscale for adolescents; SRS, Social Responsiveness Scale; SSRS, Social Skills Rating Scale; SPIB, Social and Prevocational Information Battery; SWAN, The Strengths and Weakness of ADHD symptoms and Normal behavior scale*.

## Discussion

### Summary of Main Findings

The main objective of this review was to assess the effects of digital interventions on trained skills in children and adolescents with ID. In general, the available evidence suggests that interventions undertaken with digital devices are potentially beneficial in executive function (e.g., working memory, reasoning and planning), basic cognition (as language and attention) and academic (concretely mathematics) training as well as in the social and behavioral domains. The increasing number of studies assessing the effectiveness of digital devices in the last years is noteworthy. Some studies assessed several functions at the same time, in these cases we included the same study in each of our function categories: executive functions, basic cognition skills, and behavioral and social skills (50, 63, 64). Kirk et al. (50) concluded that attention training did not improve other skills, like receptive vocabulary, phonological abilities, or cardinality. This observation suggests a possible line of research that would focus on more deeply exploring the connection between training in one skill and benefits in others.

More than a half the studies we analyzed used randomized designs, albeit most of them evaluated a small number of participants. Studies were very heterogeneous in terms of the age range, tasks and devices used. Computers were the most frequently used device in the studies, followed by handheld devices. However, we noted a lack of studies comparing the same task administered on different devices. In fact, the choice of device seemed to be adapted to the subjects' requirements. Interestingly, it has been suggested that subjects with ID perform better using a mouse than a touch screen (73), which could be attributable to motor difficulties.

Numerous tasks (mazes, puzzles, matching, discrimination, and sequences) were developed to train different functions. Although most studies used games, some used videos depicting different scenes and situations to train social skills (83, 86, 91). Several authors indicated that in order to obtain beneficial effects the tasks must include positive reinforcement, immediate feedback, and frequent repetition (45, 46, 61, 96).

Because the lengths of the sessions and the duration of the interventions varied from study to study, we did not have enough data to draw a conclusion in this regard. Two studies described positive outcomes in the social and behavioral domains after a short intervention of 2 weeks (34, 86). These studies did not include a follow-up assessment and there was not sufficient data to evaluate long-term effectiveness. We found a wide range of duration, from 4 to 27 weeks in which the effectiveness of the intervention could be demonstrated. Ottersen and Grill (47) replicated the research conducted by Söderqvist et al. (45), but they extended the length of the interventions and then compared the results of the two studies. Comparing short vs. long interventions, they concluded that progress was more constant and stable in working memory and nonverbal reasoning in long interventions.

Several studies did not specify the time at which the post-test evaluation was conducted, some did it immediately at the end of the program, while others assessed it between 1 week and 1 year after the training program ended. In general, all of the studies reported improvements when the subjects were evaluated within 1 week of training (60, 61, 64, 66, 71, 75, 78, 86, 92, 94). Only few studies included a follow-up step to assess long-term effects. Improved or maintained skills were reported at 1 and 4 months (46, 60, 64, 74, 75, 78), 5 months, (75) and at 6 months (66). A 1-year follow-up assessment did not find any effects (45), suggesting that effects could be limited in time and that a repeated intervention would be necessary to maintain the improvements achieved.

Several studies comparing digital training with traditional (or typical) interventions suggest that digital methods may be more effective than traditional ones (45–47, 50, 51, 60, 62–65, 72, 76, 86, 89, 91, 94). ID severity is an important factor to take into account, as intelligence level may limit outcomes, and subjects with moderate and severe ID will require greater support to achieve the requirements of the tasks (82). However, many authors did not specify the severity of their subjects' ID, while others combined mild and moderate ID but did not make comparisons between ID severities. Only Passig (59) observed that subjects with mild ID performed better than those with moderate ID. On the other hand, a great number of studies with subjects with mild ID reported benefits (34, 46, 62–64, 67, 72, 75, 78, 81, 85, 87, 89, 95), but few studies reported positive results in the moderate and severe ID population (65, 69, 70, 76). More studies and larger samples comparing task achievement between ID severities are needed. The majority of the studies did not specify the presence or absence of medical or psychiatric comorbidities in children with ID, but when described, the most common were genetic disorders such as Down's, Fragile X (FXS) or Williams syndromes. This is an important issue because comorbidities may add task-specific challenges. For example some difficulties were observed when training spatial knowledge in individuals with Down's syndrome using a virtual environment (96). In some cases, cerebral palsy (CP), ASD, ADHD, and motor and sensory impairment were also present. These comorbidities may interfere with task performance and the outcome of the intervention and should be noted in the results of the studies.

It is important to emphasize that new technologies do not replace the work of professionals (80), but they can help in combined interventions. Furthermore, future research is needed to assess the usefulness of other handheld devices, such as smartphones, for educational purposes. New technologies are powerful tools that can also impact the everyday lives of people with ID. The extended use of handheld devices may have beneficial effects on social and relationship skills through the construction of social networks (84) or, on the other hand, such use may have detrimental effects by increasing the risk of cyberbullying aimed at adolescents with ID (97). However, neither handheld devices nor social skills have received much attention in subjects with ID.

In agreement with a previous systematic review conducted by den Brok and Sterkenburg (42) including the ID population at all ages, we conclude that there is evidence to support the effectiveness of digital interventions in some daily living, cognitive, academic and social skills domains. However, we did not find evidence supporting long-lasting beneficial effects lasting more than a few months after the cessation of the training sessions. Moreover, to our knowledge, no research has been published that evaluates the effects of repeated interventions on long-term outcomes. In this regard, more follow-up studies are needed to examine the beneficial effects of long-term and repeated interventions.

### Limitations and Future Directions for Research

The main limitation of this review is related to the low quality of the studies analyzed, in part due to their small sample sizes. Our conclusions must be taken with caution, because monitoring the use of digital devices at home was not described and the complex characteristics of the sample could have influenced the study designs. The use of non-standardized tests and assessments rated by parents or teachers (e.g., BRIEF, ABC) was very common, and these carry a potential risk of bias due to the subjective component (46).

For future lines of research, it would be interesting to conduct studies in special education centers were training variables in training programs may be easier to control than at home. It is also necessary observe effects in subjects with severe and profound ID. It is important to consider longer follow-up assessments and longer interventions (e.g., 6 and 12 months) due to the lack of studies that make use of these longer formats, and because the improvements reported are probably not permanent (50, 78). The influence of the type of disorder giving rise to the intellectual disability as well as the most effective digital devices for use in these types of interventions should also be explored.

Despite the limitations described at the methodological level, the data analyzed suggest that digital interventions have potential as a therapeutic tool to benefit working memory, academic skills, and the social and behavioral domains in children and adolescents with ID.

## Data Availability Statement

The original contributions presented in the study are included in the article/supplementary material, further inquiries can be directed to the corresponding author/s.

## Author Contributions

Designed form, data collection, and the inclusion analysis of the studies was independently reviewed by MC and MT. When it was necessary, any disagreement was discussed with JC. All authors contributed to the study conception and design, contributed to quality analysis, commented on previous versions of the manuscript, read, and approved the final manuscript.

## Conflict of Interest

The authors declare that the research was conducted in the absence of any commercial or financial relationships that could be construed as a potential conflict of interest.

## Abbreviations

ABC: Aberrant Behavior Checklist
ADHD: Attention-Deficit/Hyperactivity Disorder
ASD: Autistic Spectrum Disorder
APA: American Psychiatric Association
AWMA: Automated Working Memory Assessment
BRIEF: Behavior Rating Inventory of Executive Function
CP: Cerebral Palsy
DSM-5: Diagnostic and Statistical Manual Fifth Edition
FXS: Fragile X Syndrome
ICT: Information and Communication Technologies
ID: Intellectual Disability
PDA: Personal Digital Assistant
PICOS: Participants, Interventions, Comparators, Outcome measures, Study design
PRISMA-P: Preferred Reporting Items for Systematic Review and Meta-Analysis Protocol.
